# How are patient inputs considered in HTA? A thematic document analysis of NICE ultra-rare disease appraisals

**DOI:** 10.1007/s10198-024-01748-1

**Published:** 2024-12-27

**Authors:** Arianna Gentilini, Alina Rana

**Affiliations:** 1 Department of Health Policy, London School of Economics and Political Science, Houghton street, London, WC2A 2AE UK; 2 Department of Economics and Public Policy, Imperial College London, London, SW7 2AZ UK

**Keywords:** Patient organisations, Rare diseases, HTA, NICE

## Abstract

Patient organisations are increasingly involved in HTA. Given this, it is important to understand what these organisations contribute and how their voices are accounted for in the decision-making process. This study characterises inputs from patient organisations and/or their nominated patient experts in technology appraisals for ultra-rare diseases in England and Wales and seeks to understand how these are considered in NICE final recommendations. We thematically analysed all HST appraisals completed between January 2022 and August 2024 (*N *= 15). We appraised inputs from patient organisations’ and experts’ written submissions, the novelty of patient inputs, as well as financial ties between contributing organisations and the manufacturer of the technology being appraised. We compared themes identified with those found in the Final Evaluation Determination documents to understand how and to what extent patients’ inputs were considered in NICE final recommendations. We found that patient submissions mainly focused on disease aspects (54%). Patients raised concerns on access challenges, caregiver burden, and mental health impacts. Most patient themes overlapped with manufacturers’ submissions (82%) and doctors’ testimonies (45%), with most novel insights focusing on access issues and mental health. Patient organisations reported receiving funding from the technology manufacturer in most appraisals, with amounts ranging from £5,000 to £74,113. Approximately half of patient inputs were explicitly mentioned in NICE final decision documents, with some considerations being neglected despite being raised by patients. While NICE incorporates many issues of importance to patients, there is room for improvement to ensure all aspects patients deem important are captured. Further research could pinpoint optimal areas for patient contributions and assess their impact.

**Supplementary Information:**

The online version contains supplementary material available at 10.1007/s10198-024-01748-1.

## Introduction

Over the past decades, patient involvement in healthcare decision-making has significantly increased, under the assumption that patients ought to have a say in decisions affecting their care [1]. In the UK, a 2020 independent review exposed how patient wellbeing in terms of drug safety and efficacy has been neglected [2, 3]. The review initiated a change in institutional prioritising and made patients a more significant stakeholder in the development and regulatory review phases, as well as in the adoption of new drugs [4–6].

One example involves the process through which decisions are made regarding which drugs are reimbursed. In this process, known as health technology assessment (HTA), a variety of stakeholders are frequently involved, from pharmaceutical companies to physicians and patients. The latter are involved under the assumption that, as intended beneficiaries of the technology appraised, they can help decision-makers understand broader considerations relating to medicines’ value [7]. Within HTA, patients are typically represented by what are known as patient organisations.

There is strong consensus in the literature that it is important to involve patients’ perspectives in HTA in order to build trust and overcome potential ethical issues [6, 8, 9]. However, there is some debate over whether patient involvement is actually relevant at the end of the R&D spectrum, as it is too late to affect crucial aspects of drug development such as clinical trial design and the selection of appropriate endpoints [10]. Some authors have also questioned whether there is an inherent conflict of interest in involving organisations that rely financially on pharmaceutical companies whose drugs are being appraised [11–13]. Others suggest that patients, longing for treatment, may tend to view new drugs favourably, regardless of the reliability of their effects [14].

Patient inputs are particularly important in the context of ultra-rare diseases, which are commonly defined as diseases that affect up to 1 person in 50,000 and are usually severe, genetically acquired and characterised by an early onset [9, 15–18]. Because of their rarity, these diseases face a number of challenges at the HTA level, notably a high degree of uncertainty around clinical benefit and quality of evidence as well as challenges in meeting standard cost-effectiveness thresholds [19–22]. In this context, patient inputs are especially important, as they can fill an evidence gap by providing insights into aspects not (or partially) captured by clinical and economic evidence [21, 23]. In some geographical settings, medicines targeting ultra-rare diseases undergo a different HTA approval process [22]. For example, in England and Wales, the HTA body, the National Institute for Health and Care Excellence, assesses medicines for ultra-rare diseases via the Highly Specialised Technology (HST) appraisal [24].

Despite the increasing involvement of patient organisations in HTA, a number of gaps remain in the literature. First, there is a high level of uncertainty around the consideration given to their submissions on HTA recommendations [25]. Second, the methodologies employed thus far to assess the consideration that patient inputs receive, such as interviews, have often proven inadequate [9, 26–29]. Lastly, no study has analysed patients’ inputs in the appraisals of medicines for ultra-rare diseases.

**Fig. 1:**
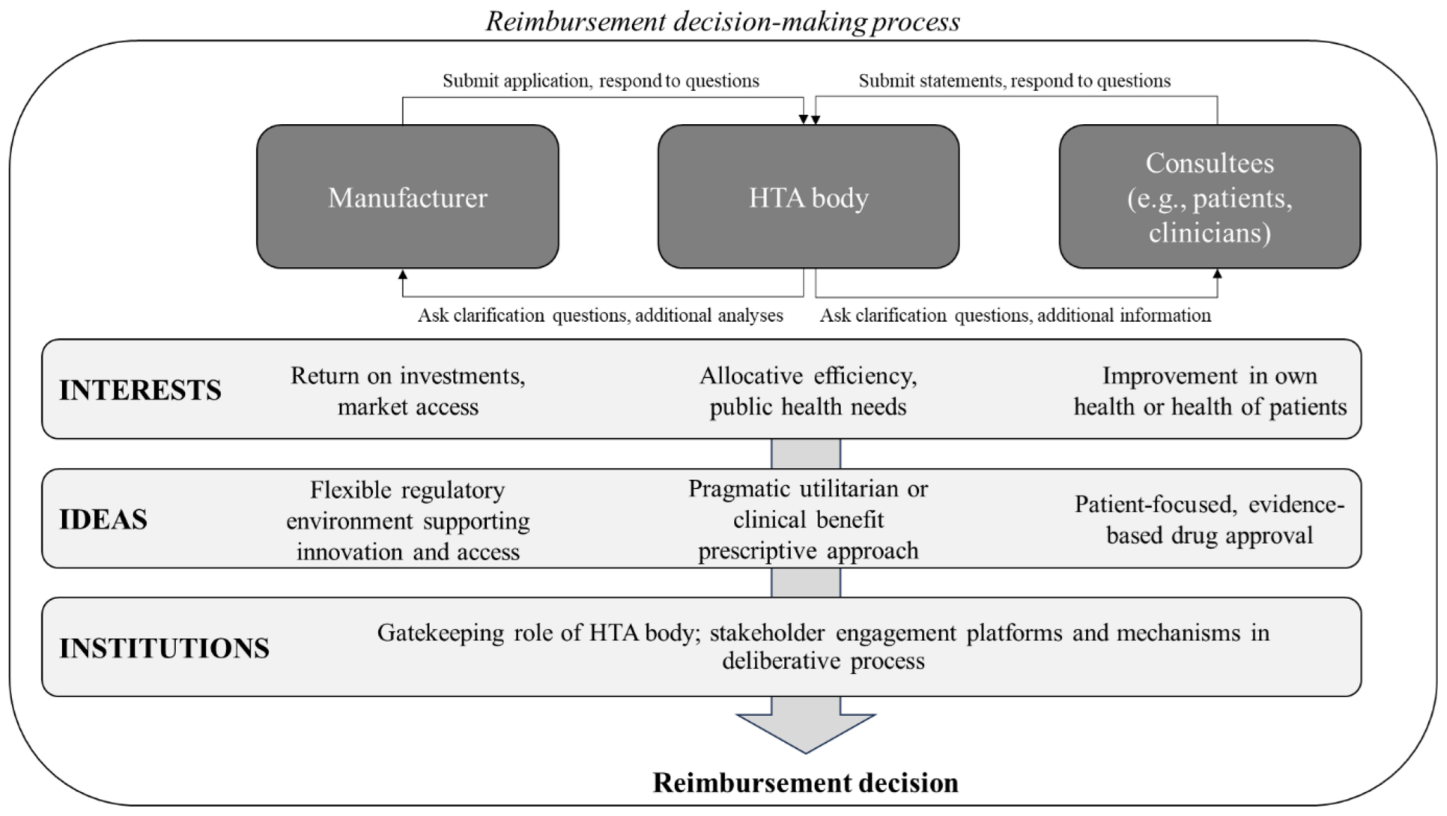
3I’s framework applied to the reimbursement decision-making process. *Abbreviations* HTA, Health Technology Assessment. *Notes* Adapted from Smith et al. (2014) [30]. Only key stakeholders are included and additional layers of complexity, including regulators and pricing dynamics, which ultimately impact access, have intentionally been omitted from this figure for simplicity. When considering interests, the HTA body, such as NICE, is expected to prioritise public health needs and allocative efficiency, while the manufacturer return on investments and market access. On the other hand, patients – either individually of as part of patient organisations – are the designated users of the technology being appraised, and, as such, have a vested interest in it being reimbursed as a vehicle to improve their own health. Similarly, clinicians are guided by the intrinsic altruistic interest of seeing their patients have access to potentially effective medicines. However, profit motives guiding doctors cannot be ruled out in the case of conflicts of interests. Turning to ideas, some HTA agencies, such as NICE, might be guided by a pragmatic cost-effectiveness utilitarian approach, while others might focus on different priorities, such as clinical benefit [32]. Nevertheless, they are broadly guided by a prescriptive approach positing that they act in the interest of the public by addressing information asymmetries between innovators and users of medicines. Manufacturers, on the other hand, advocate for a more flexible regulatory environment supporting innovation and access, which, for example, translates into less stringent evidence requirements to demonstrate the efficacy of their products, such as a wider use of surrogate endpoints and of phase II trials [33]. Patient organisations and experts may push for broader inclusion of patient perspectives and qualitative evidence in HTA, while clinicians are expected to uphold evidence-based approvals. Finally, an example of the *institutions* domain is the gatekeeping role of NICE to the English and Welsh healthcare market, with approximately 60 million potential consumers, which confers notable power to the body [34]. In fact, NICE recommendations are binding, and the National Health Service (NHS) is legally obliged to fund and resource medicines and treatments NICE recommends [35]. Similarly, stakeholder engagement platforms and mechanisms, such as public consultations and expert committees, provide avenues for actors to influence HTA processes within established institutional frameworks

This paper builds on the literature on patient involvement in HTA to address these gaps and look at how patient organisations and experts representing them figure in this process. First, we look at what patient organisations and their nominated experts contribute to the NICE HST appraisals, assessing the uniqueness of their inputs compared to other stakeholders and identifying any financial ties with the manufacturer of the technology under review. Second, we analyse how patients’ inputs are considered by decisionmakers by exploring to what extent these are taken into account in final NICE recommendations, included in the Final Evaluation Document (FED). Figure 1 illustrates the conceptual framework that underpins the analysis, namely the ‘3I’s’ framework of interests, ideas and institutions [30]. This provides us with a lens through which to make sense of the findings and enables us to characterise the dynamics at play between the various actors involved in the appraisal process. When considering HTA processes in a single-payer healthcare system, there are usually two main actors, namely the HTA body, which is typically in charge of advising the government on whether to reimburse a technology or not, and the pharmaceutical company manufacturing it, wishing to gain access to a certain market. However, in deliberative HTA processes, such as the one adopted by NICE, other stakeholders are consulted, including professional groups, clinicians and patient organisations and experts. Broadly speaking, interests refer to how stakeholders pursue their personal or collective goals in the promotion of policy decision (e.g., return on investments), ideas are defined as ‘values and beliefs through which individuals make sense of the world’ (e.g., prescriptive approach), while institutions include the laws, regulations, and procedural norms that shape processes (e.g., stakeholder engagement platforms). While discussed individually, these dimensions are interdependent and jointly determine the outcome of the decision-making process.

This paper contributes to the literature in three main ways. First, this is the first paper of its kind to unpack patient contributions to HTA appraisals in ultra-rare diseases. Second, this is also the first paper to tackle this topic using document analysis, which has the potential to overcome methodological issues around the difference between stated and actual behaviours, a common issue in interviews. Finally, we contribute to the existing literature by expanding the conceptual understanding of the dynamics between patient organisations, experts, NICE, and manufacturers during the reimbursement decision-making process. NICE was purposively selected as it has a longstanding history of patient involvement, especially in the context of rare diseases, and HST appraisals were chosen as they provide a valuable case study for assessing ultra-rare disease and ensure consistency in document analysis [29, 31].

The rest of the paper is structured as follows. The Literature review section summarises the evidence on the topic of patient involvement in HTA. The Methods and Results sections describe, respectively, the methodology and the documents used in the analysis and the study results. Finally, the Discussion section concludes and discusses policy implications.

## Literature review

There is a growing literature focusing on the involvement of patients’ voices in HTA. Studies can be broadly categorised in three groups: motives for patient involvement in the HTA process (or lack thereof), ways in which this involvement take place, and the assessment of their engagement.

A number of reasons have been laid out as to why patients’ perspectives in HTA should be included in HTA [8, 9, 36]. Wale and colleagues argue that according to the Alma-Ata Declaration, patients have the right to participate in the planning and delivery of their health care [37]. Furthermore, they can provide valuable insights to inform HTA decision-making, including their experience with the condition, treatment, and unmet needs. Patient involvement in the HTA process can lead to better policy outcomes, greater transparency, and accountability, and increased public trust in the health system [6, 37]. Finally, some argue that a key motivation in adopting public and patient involvement in drug assessment is to bolster the democratic legitimacy of the HTA process [38]. Conversely, some authors have pointed out issues that might make patients’ involvement in HTA flawed and offer little benefit. Edwards points out that, if patients are consulted at the end of the R&D spectrum, they are unlikely to affect crucial aspects of drug development such as clinical trial design and the selection of appropriate patient-reported outcome measures (PROMs) [10]. On a similar note, Lynch and Largent discuss how current patients who are sick today understandably tend to view new promising drugs favourably, regardless of the rigor of their trial design and the reliability of their effects [14]. This is consistent with the findings of a Canadian study, which found that patient organisations providing inputs to funding decisions almost always expressed a positive view on the technology under consideration, regardless of whether they had financial ties with the company making the product [39]. Additionally, patient organisations and patient experts participating in HTA appraisals face significant opportunity-costs, such as time and financial resources. As a result, only particularly motivated or well-funded individuals or organisations tend to take part in this process [6, 8, 25, 40]. Such self-selection can have important equity implications, with certain disease areas being underserved in terms of representation in HTA processes and other stages of research and development [8, 12, 41]. Others expressed concern around their financial dependency on industry funding [11–13]. In a study by Barnes and colleagues, committee members expressed their distrust in patient inputs due to potential bias and the representation of views from pharmaceutical companies instead of genuine opinions [42]. Finally, some authors highlighted the fact that patients’ testimonies are usually subjective and experiential, casting doubts on whether they are reflective of the entire patient population they wish to represent, and that they likely offer further context and personal insights into the clinical and economic evidence already presented by other stakeholders, rather than introducing entirely novel perspectives [9, 26, 36, 43].

The involvement of patient organisations and experts in the HTA process varies across jurisdictions, influenced by cultural, political, and historical factors [44]. Most HTA bodies elicit patients’ perspectives during the appraisal stage, with the exception of NICE, where patient organisations and experts are also involved in the scoping stage of the appraisals [45]. Patient inputs are commonly delivered in the form of written submissions to the HTA committee, but they can also take the form of statements made in public meetings or hearings, and comments on final recommendations. Additionally, every jurisdiction involves patients differently. For instance, to enhance patient representation, in 2017, the Haute Autorité de Santé – the French HTA body – created an online tool to collect data related to inputs from patient organisations, while in England and Wales, NICE involves a handful of patient groups and experts [8]. Finally, the degree to which patient involvement is institutionalised also varies considerably. While NICE has a clear pathway for how patient organisations and experts can submit statements and participate in committee meetings, in Germany, the G-BA accepts submissions from any external stakeholder, regardless of whether they are patients or not [43].

Lastly, the literature assessing patients’ involvement in HTA processes is modest. Hamilton and colleagues attempted to quantitatively assess whether patient inputs affected final HTA coverage decisions by comparing the proportions of technologies accepted, accepted with restrictions and not recommended with and without submissions from patient groups [29]. Similarly, Chang et al. estimated the association between patient groups’ submissions and positive reimbursement decisions from both NICE and the Sottish Medicines Consortium, the Scottish HTA body, finding no statistically significant results [28]. However, as acknowledged by the authors themselves, this approach is likely to overlook several important factors that might contribute to coverage decisions [28, 29]. Other studies have used interviews to understand how decision-makers incorporate patient views [9, 26, 27]. While allowing a more granular analysis, interviews can be prone to bias, as interviewees may be part of the HTA committee and hold preconceived notions regarding how patient views should be taken into account. As insiders to the system, they may be less likely to describe patient inputs as tokenistic. Furthermore, a barrier to using stated preference from interviews results in decision-making is that the preferences stated may not predict actual behaviour [46]. More specifically, people tend to overstate their preferences for so-called moral goods or attitudes that show social responsibility [47, 48].

## Methods

### Study design

The chosen design for this study is a document analysis. We thematically analysed the scope, frequency, and intensity of patient inputs, defined as inputs from patient organisations and their nominated experts, associated with 15 HST appraisals completed between January 2022 and August 2024. This timeframe was purposively selected because it encompasses more than 50% of the 28 appraisals since the program’s inception in 2013, providing a recent snapshot of how NICE incorporates patients into its ultra-rare diseases appraisals [49]. This selection also includes all drugs assessed since the publication of NICE’s updated manual for its four health technology evaluations, including the HST pathway, in January 2022 [50].

Patient inputs were categorised according to themes following a deductive/inductive approach. As part of the deductive component, we reviewed the HTA literature to identify papers and themes pertaining to patient inputs or rare disease assessment. Two studies, Berglas and colleagues and Nicod and Kanavos, met these criteria [23, 51]. The former study analyses assessments by the Canadian Agency for Drugs and Technologies in Health (CADTH) Drug Expert Committee to understand whether and to which extent patient groups’ insights are taken into account, while the latter develops a framework and identifies key factors that influence coverage decisions of orphan drugs – intended as drugs for rare diseases – in HTA. These two frameworks were used as a starting point for iterative inductive coding of patient inputs, which began with the most recent assessment and worked backward, until no new theme could be identified (i.e., saturation). Identified themes were nested within broader categories, following a tiered system. A higher tier indicates increasing specificity of the themes to reflect the evidence available from the documents. Both tier 1 and 2 themes can be either disease-, technology-, or submission-specific.

Table 1 illustrates the coding structure. *Disease-specific* themes refer to how it is to live or care for someone living with the condition (e.g., quality of life, unmet need), while *technology-specific* themes relate to patient experts’ view on the technology being appraised (e.g., impact on symptoms). To ensure completeness of the study, we also included *submission-specific* considerations from patients. These include comments from patient organisations and experts on the clinical and economic evidence for the technology appraised. It is important to note that submission specific considerations are included in the table because they are explicitly mentioned in the FED but are unlikely to be reported in committee papers in the initial submission from patient organisations and experts, as these are usually raised in later oral consultation or technical responses. Because of their low frequency, *submission-specific* inputs are not broken down in two tiers.

**Table 1:** Coding structure

Umbrella theme	Tier 1 theme	Tier 2 theme	Description	Examples
Disease-specific	Treatment options	Unmet need	Lack of or few treatments available alternatives available	“There is a clear unmet need for this technology. There no treatment option only best supportive care”
		Suboptimal treatment pathway	Issues about current treatment pathways, such as the fact that treatment options are invasive, associated with many complications or simply not routinely available	“Surgeries carry increased risk to patients and are avoided where possible”
		Access	Access considerations, such as equality, socioeconomic barriers, access to relevant health services, need-based allocation, and benefits according to patient subtypes	“We believe that denying children the opportunity of a proven lifesaving treatment would demonstrate Inequality and inequity”
	Current quality of life	Physical disabilities	Living with the conditions is associated with physical issues that make daily activities complicated	“Children rapidly lose the ability to walk, talk, swallow, see, hear and become incontinent; they develop serious muscular and skeletal complications”
		Mental health	Living with the condition is associated with mental and emotional struggles such as anxiety, depression etc.	“Mental health issues are abundant in affected families”
		Daily life, social life, and education	Living with the condition is associated with struggles in daily life activities which are not problematic for healthy people, limited social interactions and impacts education opportunities such as having friends, going to college etc.	“Due to the extensive range of symptoms and difficulties experienced by patients, it soon becomes impractical for the majority of children to attend school”
		Carer burden	Physical, emotional, and psychological stress that carers face in the carer role, which can have a significant impact on their overall well-being and quality of life	“Parents have also communicated the physical implications of caring for their child, due to manual handling, including tendinitis, neck pain, back pain, shoulder pain and hip pain”
Technology-specific	Quality of life with technology	Independence	No longer dependent upon a caregiver to receive treatment or for basic self-care	“100% of treated patients are able to walk independently”
		Symptoms	Change in specific symptoms, such as fatigue, seizure frequency, attack severity, ability to breathe, eat, sleep, or move	“No pain and muscular skeletal issues in treated patients”
		Mental health	Change in mental health-related aspects such as confidence, emotional wellbeing	“Another significant mark of success for us has been […] the positive effect which the opportunity of treatment has had on the patient psyche and mental health”
		Cost	Change in the cost borne by individual patients in accessing treatment	“The biggest disadvantage of the treatment was the fact we had to travel abroad […]. There were obvious cost implications in having to do this, flights, taxi’s, etc.”
	Administration	Frequency of administration	Number and frequency of pills or injections that might affect the ability and willingness to continue taking medication	“Gene therapy itself is a fairly straightforward one-off procedure”
		Ease of administration	Mode of administration of the technology, such as pills or subcutaneous/intravenous injection	“[The drug] is not a pill. It is spending four hours a week hooked up to a drip”
	Safety	Adverse events	Side effects of the treatment	“A small number of children suffered from an infection during the period of reduced immune system”
Submission-specific	Clinical evidence	The comments pertain the company’s submission, NICE or ERG comments regarding the clinical evidence related to the technology assessed (e.g., clinical outcomes in trial etc.)		“These are important outcomes for patients, however the Beck depression inventory is a very poor tool for measuring outcomes as it is not a balanced measure of mental health or mood”
	Economic evidence	The comments pertain the company’s submission, NICE or ERG comments regarding the economic evidence related to the technology assessed (e.g., economic modelling, utility values used etc.)		“From the perspective of the economic model, this is primarily due to the challenge of finding quality of life indicators that are measurable within the confines of the economic model”

*Abbreviations* ERG, Evidence Review Group; NICE, National Institute for Health and Care Excellence

Next, we compared the themes identified in patient organisations and experts’ written submissions in support of HST appraisals with those found in the FEDs to understand whether and to which extent patients’ inputs were considered in NICE final recommendations. To do this, we followed the methodology proposed by Barlow and colleagues, who examined the impact of industry on global alcohol policies [52]. Specifically, we looked for any instances where the committee explicitly stated that their considerations reflected points raised by patient organisations and patient experts. Two authors (AR, AG) coded the data, and one author (AG) blindly re-coded a 30% random sample of the HST appraisals to validate the coding from the second authors (AR). Any disagreement was discussed until consensus was reached.

The data collected was analysed to assess the following outcomes of interest: (1) the type, frequency and intensity of themes patients contributed to; (2) novelty of patient inputs versus inputs from other stakeholders; (3) self-declared financial ties between organisations providing submissions and manufacturers of the technology under appraisal and/or comparators; (4) whether and to what extent patient inputs were explicitly mentioned in the FED. The type, frequency and intensity of patient inputs in HST appraisals illustrate the issues that are of highest importance to patient organisations and experts. Linking to the conceptual framework discussed above (Fig. 1), patient inputs are expected to indicate their *ideas*. Importantly, we also looked at whether themes raised by patients have been discussed by other stakeholders, namely, manufacturers and doctors, in individual capacity or as part of a professional group. While we cannot make definitive statements on whether restating issues has a different impact than presenting novel ones, documenting whether patient inputs provide new evidence or not can help us understand the areas where patient inputs are likely to have the biggest impact and whether they are aligned with specific stakeholders (and *interests*). This information was extracted from manufacturers’ submissions and written statements by doctors and professional organisations through keyword searches. For instance, if patients expressed concerns about the challenges faced by children affected by the disease in attending school, searches involving terms like “education” or “school” were conducted. Similarly, if patients lamented the lack of treatment options, keywords like “unmet need” were used in the search.

An issue emerging from the literature review was the potential conflicts of interest between patient organisations providing testimonies as part of the technologies’ appraisals and pharmaceutical companies, which might affect their impartiality [11, 53]. To explore this, we collected the patient organisations’ disclosure statements included in NICE committee papers, where the NICE submission form (question 4b) asks whether the organisation received any funding from the manufacturers of the technology and/or comparator products in the 12 months preceding the submission. This analysis allowed us to better understand the effectiveness of NICE’s disclosure policies and assess whether contributing organisations may have conflicts of interest related to the technology or disease area under evaluation.

The recurrence of themes in the FED aims to shed light on which aspects, if any, patients are more likely to be perceived as most relevant by the NICE committee in the context of ultra- rare diseases in England and Wales.

The analysis also presents descriptive statistics including the disease type, the age of onset, the number of patient groups and experts contributing to the HST appraisals in scope as well as whether the patient experts were nominated by patient organisations. Finally, to provide further context on the technologies being appraised, we document key clinical evidence, such as trial design, phase, and primary endpoint(s). We also report on the clinical benefit assessment ratings given to the drugs in the sample by other HTA bodies, namely the German Federal Joint Committee (G-BA) and the French Haute Autorité de Santé (HAS), to shed light on their clinical value in the therapeutic pathway.

### Document selection

This analysis focuses on the initial written submissions from patient organisations and their nominated experts during the guidance development phase of HST appraisals, which are included in NICE’s first committee papers. Initial written submissions were chosen as this is where patient organisations and experts can highlight their views on the technology being appraised, and, conversely from oral consultations and responses to comments, they follow a predetermined structure which allows homogeneity of analysis [54]. Information on payments to organisations submitting statements as part of the appraisal process was also retrieved from the initial committee papers, specifically from question 4b, where NICE asks organisations: “Has the organisation received any funding from the manufacturer(s) of the technology and/or comparator products in the last 12 months?”. Further details on how NICE involves patients in its appraisals can be found in Appendix A.

As part of this study, the FED for each HST appraisal is also reviewed, which present the committee’s final recommendations regarding the use of highly specialised medicines in England and Wales. For recently published guidelines (HST 28, HST 30 and HST 31) FED were not available, so final draft guidances were used instead. Analysing these documents enable us to examine whether and to which extent patient inputs are considered in NICE’s final recommendations. All documents are publicly available from NICE’s website. Data sources consist of the first committee papers and FEDs, which are publicly available on the NICE website in the *history* section of each HST appraisal. Links for the where to find the documents analysed can be found in Appendix C.

Documents were downloaded in PDF format throughout August 2024, and relevant sections were highlighted. These highlighted portions were also recorded in a data extraction sheet and analysed in Excel. Links to all documents from which data were extracted are available in Appendix C.

## Results

### Descriptive statistics

Between January 2022 and August 2024, 15 drugs have been assessed via the HST pathway and, therefore, are included in the analysis. All technologies but afamelanotide (HST 27), which received a negative reimbursement opinion, were approved, either for their entire marketing authorisation label or a subset of the licensed population. Table 2 illustrates the characteristics of technologies assessed via the HST pathway. Most of the technologies appraised target conditions affecting infants or children, with a smaller subset also addressing adults. Out of the 15 appraisals analysed, three involved the re-evaluation of existing guidelines, primarily presenting results from real-world evidence studies on clinical effectiveness. The majority of HST appraisals had at least one randomised controlled study supporting the manufacturer’s submission, but single-arm trials were also common for ethical reasons, primarily related to the issue of withholding treatment from severely ill patients [55]. In cases with a comparator arm, this was non-active, comprising of best supportive care or off-label medicines. For single-arm trials, comparative effectiveness was based on data from natural history cohorts (i.e., registries). The number of patients enrolled in the main clinical trials supporting the manufacturers’ submission varied from 9 to 350, with higher number collected via observational studies, also known as real-world studies. All primary endpoints except for asfotase alfa (HST 23) were surrogate, meaning that they are not clinically meaningful endpoints, such as survival, but are assumed to correlate with them [56]. All drugs included in the analysis were assessed in France and Germany, with available GBA and HAS reports (see Appendix C for further details). Most G-BA ratings were of non-quantifiable or minor additional benefit compared to existing treatment alternatives. Similarly, according to HAS most drugs show important clinical benefit (SMR Important), but varying levels of added improvement (ASMR II to V), indicating a mix of significant to no added value compared to existing treatment alternatives.

The number of patient organisations participating in the first committee meetings and submitting their written testimonies ranged from zero to two, while for patient experts it ranged from zero to three. With the exception of the setmelanotide and afamelanotide appraisals (HST 21 and HST 27), which did not have any written submission from patient organisations and patient experts, respectively, all patient experts were nominated by patient organisations. Most patient organisations contributed to a single appraisal. However, some patient organisations participated to multiple appraisals, likely due to the broader scope of diseases supported. For example, The MPS Society and Metabolic Support UK participated in four and three different appraisals, respectively, followed by Muscular Dystrophy UK, which provided an organisational written submission for two appraisals.

**Table 2:** Characteristics of HST appraisalsanalysed

**HST #**	**HST 17**	**HST 18**	**HST 19**	**HST 20**	**HST 21**	**HST 22**	**HST 23**	**HST 24**
INN	Odevixibat	Atidarsagene autotemcel	Elosulfase alfa	Selumetinib	Setmelanotide	Ataluren	Asfotase alfa	Onasemnogene abeparvovec
Brand name	Bylvay	Libmeldy	Vimizim	Koselugo	Imcivree	Translarna	Strensiq	Zolgensma
Manufacturer	Albireo AB	Orchard Therapeutics	BioMarin	AstraZeneca	Rhythm Pharmaceuticals	PTC Therapeutics Limited	Alexion	Novartis
Approved	Yes, with confidential commercial discount							
Date of HST guidance publication	22/02/2022	28/03/2022	20/04/2022	05/05/2022	06/07/2022	22/02/2023	01/03/2023	19/04/2023
New or re-evaluated HST guidance	New	New	Re-evaluation	New	New	Re-evaluation	Re-evaluation	New
Disease type	Progressive familial intrahepatic cholestasis	Metachromatic leukodystrophy	Mucopolysaccharidosis type 4 A	Neurofibromatosis type 1-associated plexiform neurofibromas	Obesity caused by LEPR or POMC deficiency	Duchenne muscular dystrophy with a nonsense mutation in the dystrophin gene	Paediatric-onset hypophosphatasia	Presymptomatic spinal muscular atrophy
Disease onset	Infancy, childhood, young adulthood	Late infantile, juvenile	Early childhood	Early childhood	Early childhood	Infancy to adulthood	Perinatal to adulthood	Perinatal to adulthood
Clinical evidence*								
Study design	Phase III RCT and open-label extension study	Phase II non-randomised, open-label, prospective, single-centre trial	Phase III RCT and open-label extension study	Phase II single-arm, open-label trial	2 phase III, single-arm open-label trials and open-label extension studies	2 phase III RCT, 2 real-world studies	4 open-label phase II studies of which 2 RCT and 2 non-RCT; 2 extension studies	Phase III, single-arm open-label trial
Trial comparator	Placebo	None	Placebo	None	None	Placebo (RCT)	Placebo (for one RCT)	None
Num. of patients	62 (RCT); 71 (open-label extension)	20	173 (RCT); 169 (open-label extension)	50	30 (phase III studies), 15 (extension study)	307 (RWE); 230 (RCT)	34 (RCT), 80 (non-RCT)	29
Primary endpoint(s)	Reduction of at least 70% in serum bile acid level from baseline at 24 weeks vs. placebo	Improvement of at least 10% in total GMFM score vs. natural history cohort on BSC; Statistically significant increase in residual ARSA enzyme activity by at least 2 standard deviations vs. pre-treatment values	6 min walk test (6MWT) of between 30 m and 325 m vs. natural history cohort on BSC	Rate of confirmed partial response and complete response using centrally read volumetric MRI vs. natural history cohort on BSC	Proportion of people having at least a 10% weight loss with setmelanotide from baseline to 52 weeks	6MWT at week 48 vs. placebo	OS; Ventilator-free survival vs. natural history cohort on best supportive care	Sitting without support for at least 30 s; Standing alone for at least 3 s vs. natural history cohort on best supportive care
G-BA rating**	Minor additional benefit	Late infantile (LI) or early juvenile (EJ) forms of MLD without clinical manifestations: Major additional benefit EJ form of MLD with early clinical manifestations: Non-quantifiable additional benefit	Minor additional benefit	Non-quantifiable additional benefit	Non-quantifiable additional benefit	Minor additional benefit	Non-quantifiable additional benefit	Additional benefit not proven (sales exceeding > 50 million EUR)
HAS rating**	SMR Important ASMR III	(LI) or early juvenile (EJ) forms of MLD without clinical manifestations: SMR Important EJ form of MLD with early clinical manifestations SMR Insufficient ASMR III	SMR Important ASMR IV	SMR Important ASMR IV	SMR Important ASMR IV	SMR Faible ASMR V	SMR Important ASMR II	Patients with SMA Type 1 or 2: SMR Important Patients with SMA Type 3: SMR Insufficient ASMR III
**Patient inputs**								
No. of POs	1	3	2	1	0	2	1	1
POs	Children’s liver disease foundation	ArchAngel MLD Trust; MLD Support Association UK; The MPS Society	Rare disease research partners; The MPS Society	Childhood Tumour Trust	N/A	Muscular Dystrophy UK; Action Duchenne	Metabolic Support UK	Muscular Dystrophy UK; Spinal Muscular Atrophy UK
Funding from manufacturer of the technology and/or competitors in 12 months before submission	£25,988 (Albireo) £13,914 (Mirium pharmaceuticals)	ArchAngel MLD Trust: £5,000 (Orchard Therapeutics) MLD Support Association UK: £7,250 (Orchard Therapeutics) The MPS Society: £14,000 (Orchard Therapeutics) MLD Support Association UK & The MPS Society: £11,600 (Orchard Therapeutics)	Rare disease research partners: RDRP has received fees for professional services provided to BioMarin The MPS Society: £56,000 (BioMarin)	Declared no funding from the tobacco industry†	N/A	Muscular Dystrophy UK: £64,412 (PTC Therapeutics) Action Duchenne: £60,000 (PTC Therapeutics)	£10,887.5 (Alexion)	Muscular Dystrophy UK: £5,000 (Novartis) Spinal Muscular Atrophy UK: £74,112.83 (Novartis)
No. of PEs for WS	1	3	3	2	2	2	1	2
Are PEs nominated by POs?	Yes	Yes	Yes	Yes	Yes	Yes	Yes	Yes
**HST #**	**HST25**	**HST26**	**HST27**	**HST 28**	**HST 29**	**HST 30**	**HST 31**	
INN	Lumasiran	Eladocagene exuparvovec	Afamelanotide	Birch bark extract	Velmanase alfa	Sebelipase alfa	Setmelanotide	
Brand name	Oxlumo	Upstaza	Scenesse	Filsuvez	Lamzede	Kanuma	Imcivree	
Manufacturer	Alnylam	PTC Therapeutics Limited	Clinuvel	Chiesi✽	Chiesi	Alexion	Rhythm Pharmaceuticals	
Approved	Yes, with confidential commercial discount		No	Yes, with confidential commercial discount				
Date of HST guidance publication	19/04/2023	19/04/2023	26/07/2023	20/09/2023	13/12/2023	10/01/2024	22/05/2024	
New or re-evaluated HST guidance	New	New	New	New	New	New	New	
Disease type	Primary hyperoxaluria type 1	Aromatic Lamino acid decarboxylase deficiency	Erythropoietic protoporphyria	Dystrophic and junctional epidermolysis bullosa	Mild to moderate alphamannosidosis	Wolman disease	Obesity and hyperphagia in Bardet-Biedl syndrome	
Disease onset	Infancy to adulthood	Childhood	Adulthood	Infancy to adulthood	Infancy	Infancy	Childhood to adolescence	
Clinical evidence*								
Study design	Phase III RCT, with open-label extension study	3 phase III single-arm open label trials	4 phase III double-blind RCT	Phase III double-blind RCT	Phase III RCT, with single-arm open-label extension study	1 phase II, 1 phase II/III single-arm RCT, and a natural history study	Phase III single-arm RCT, with open-label extension study	
Trial comparator	Placebo	None	Placebo	Placebo (control gel)	Placebo (RCT)	None	None	
Num. of patients	39 (RCT and extension study)	28	350	223	25 (RCT), 33 (extension study)	10 (phase II), 9 (phase II/III), 35 (natural history study)	38 (RCT and extension study)	
Primary endpoint(s)	Percentage change in 24 h urinary oxalate excretion from baseline to month 6 vs. best supportive care	Proportion of people who reached the key motor milestones as per the PDMS-2 scale vs. natural history cohort on best supportive care	Sun exposure (pain-free direct sunlight between 10:00 and 15:00 h or 18:00)	Proportion of people with a first complete target wound closure within 45 days	Change from baseline to month 12 in serum oligosaccharides Change from baseline to month 12 in the 3-MSCT	Severe treatment-emergent adverse events (phase II) Proportion of people alive at 12 months old (phase III)	Proportion of patients aged ≥ 12 years who achieved at least10% bodyweight reduction from baseline after 52 weeks	
GBA rating**	Non-quantifiable additional benefit	Non-quantifiable additional benefit	Non-quantifiable additional benefit	Minor additional benefit	Non-quantifiable additional beneft	Non-quantifiable additional benefit	Non-quantifiable additional benefit	
HAS rating**	SMR Important ASMR III	SMR Important ASMR III	SMR Important ASMR IV	Dystrophic EB: SMR Faible Other indications under MA: SMR Insufficient ASMR V	SMR Important ASMR IV	Long-term ERT in rapidly progressive forms of LAL deficiency starting in infancy: SMR Important ASMR III Other forms of LAL: SMR Faible ASMR V	SMR Important ASMR IV	
**Patient inputs**								
No. of POs	1	2	1	1	1	2	1	
POs	Metabolic Support UK	The AADC Research Trust; Metabolic Support UK	British Porphyria Association	DEBRA UK	The MPS Society	Children’s Liver Disease Foundation; The MPS Society	Bardet-Biedl Syndrome UK	
Funding from manufacturer of the technology and/or competitors in 12 months before submission	Declared no funding from the tobacco industry†	Declared no funding from the tobacco industry†	Declared no funding from the tobacco industry†	£71,000 (Amryt†)	Declared no funding from the tobacco industry†	Children’s Liver Disease Foundation: No funding received The MPS Society: £38,800 (Alexion)	£7,000 (Rhythm Pharmaceuticals)	
No. of PEs for WS	0	1	0	2	1	2	2	
Are PEs nominated by POs?	N/A	Yes	N/A	Yes	Yes	Yes	Yes	

*Abbreviations* 6MWT, 6 min walk test; ARSA, Arylsulfatase A, BSC, Best supportive care; CP, Committee papers; EB, Epidermolysis bullosa; ERT, Enzyme replacement therapy; GMFM, Gross Motor Function Measure; INN, International Non-proprietary Name; LAL, Lysosomal acid lipase; LEPR, Leptin receptor; MA, Marketing authorisation; MLD, Metachromatic leukodystrophy; MRI, Magnetic Resonance Imaging; MSCT, Modified Schirmer’s Tear Test; OS, Overall survival; SMA, Spinal muscular atrophy; PDMS-2, Peabody Developmental Motor Scales Second Edition; PE, Patient expert; PO, Patient organisation; POMC, Proopiomelanocortin; RCT, Randomised controlled trial; WS, written submission

* Clinical evidence data in the table refer to the main clinical studies supporting the manufacturers’ reimbursement application, rather than all evidence for the technology assessed

** The GBA ratings include six categories that indicate the added benefit of a technology compared to the standard of care (SoC). These are: major added benefit (substantial additional benefit over SoC), considerable added benefit (moderate benefit over SoC), minor added benefit (slight improvement compared to SoC), non-quantifiable benefit (benefit exists but cannot be quantified due to insufficient evidence), no added benefit (no improvement compared to existing treatments), and less benefit (inferior to the existing treatment). These ratings influence drug pricing and reimbursement decisions in Germany, with higher ratings leading to better pricing outcomes

HAS evaluates treatments through two main ratings: the SMR (Service Médical Rendu), which measures the clinical benefit, and the ASMR (Amélioration du Service Médical Rendu), which assesses the added clinical value compared to alternatives. The SMR ratings range from major to insufficient clinical benefit, while the ASMR ratings span from major improvement (ASMR I) to no improvement (ASMR V). The ASMR rating plays a crucial role in determining reimbursement levels and pricing, with higher ASMR scores leading to more favourable reimbursement conditions [32]. Different G-BA and SMR ratings are reported when they vary between subpopulations included in the technology’s label

† In organisational submissions for HST 20, 25, 26, 27, and 29, the NICE form did not include the question “Has the organisation received any funding from the manufacturer(s) of the technology and/or comparator products in the last 12 months?” as question 4b. Instead, it asked, “Do you have any direct or indirect links with, or funding from, the tobacco industry?” For those appraisals, the table reports whether the patient organisation declared any links with or funding from the tobacco industry

The NICE submission was initially filed by Amryt, which was then acquired by Chiesi in 2023

### Financial ties between organisations providing submissions and manufacturers of the technology under appraisal and/or comparators

To assess financial ties between manufacturers and patient organisations providing submissions to NICE, we analysed self-reported disclosures regarding funding received from manufacturers of the technology under appraisal and/or comparator products in the 12 months preceding submission, as reported in NICE submission forms. The amounts of funding disclosed across the 15 appraisals ranged from £5,000 to £74,112.83 (Table 2). While the question explicitly asked for information on funding from both the manufacturer of the technology being assessed and its competitors, only one organisation, the Children’s Liver Disease Foundation (HST 17), reported receiving funding from a competitor.

The highest manufacturer funding was reported in ataluren’s appraisal (HST 22), where Muscular Dystrophy UK and Action Duchenne respectively disclosed receiving £64,412 and £60,000 from PTC Therapeutics. The highest single funding reported was in the appraisal for onasemnogene abeparvovec (HST 24), where Spinal Muscular Atrophy UK disclosed receiving £74,113 from Novartis. This was followed by the appraisal of birch bark extract (HST 28), where DEBRA UK reported receiving £71,000 from Amryt, the company initially responsible for product development and the NICE submission, before being acquired by Chiesi in 2023. Other organisations reported smaller amounts, such as £5,000 received by ArchAngel MLD Trust from Orchard Therapeutics, and £7,250 received by the MLD Support Association UK from the same company in atidarsagene autotemcel’s appraisal (HST 18).

The funding was attributed to supporting a range of activities, such as organising conferences, setting up real-world registries, research grants, or providing general support during the COVID-19 pandemic. However, the level of detail provided about these activities varied across the reporting from patient organisations.

In one third of the appraisals, the wording in the NICE form differed, with patient organisations not being asked about financial ties to the pharmaceutical company in question 4b of the submission template. Instead, they were asked if they had any direct or indirect links with, or funding from, the tobacco industry (specifically: ‘Do you have any direct or indirect links with, or funding from, the tobacco industry?‘). All organisations reported having no such links.

### Types and frequency of patient inputs in written submissions

A total of 644 unique patient inputs – intended as theme-specific statements from both patient organisations and experts – were identified in their written submissions in support of the 15 HST appraisals assessed (Table 3). Disease-specific themes were more prevalent than technology-specific ones, accounting for 345 (54%) and 237 (37%) of all themes raised. The remaining 62 (10%) focused on comments related to the company submission.

When looking at tier 1 themes, *current quality of life* was the most frequently discussed in patients’ written submissions (*N* = 185; 29%), followed by *treatment options* (*N* = 160; 25%), and *quality of life with technology* (*N* = 148; 23%). The most mentioned tier 2 themes were *symptoms*, *unmet need*, *physical disabilities* and *carer burden*, each being raised in 10% or 9% of inputs overall. Patients’ statements highlighted the lack of treatment options, the physical difficulties faced in everyday activities while living with the disease, the improvement in symptomatic manifestations due to treatment, and difficulty providing care for the child in the absence of institutional supports such as care staff at home.

Issues related to *suboptimal treatment pathway* (*N* = 53; 8%) were also frequently raised. Patients explained difficulties in accessing support, getting timely diagnosis, and the lack of awareness of conditions amongst the healthcare professionals. They also discussed the complexity in existing treatment pathways, including multiple, invasive treatments or those with significant side effects add to the disease burden. Patient organisations and experts also highlighted structural barriers in accessing medical and non-medical treatment which increased the financial difficulties. Mental health issues from the stress of dealing with the condition for both carers and patients alike were amongst the concerns raised. Interestingly, *clinical evidence* (*N* = 54; 8%) was also frequently discussed by patient organisations and experts, who raised questions about the limited data on long term efficacy of the treatments being considered and the overwhelming reliance on data from clinical trials instead of real-world-evidence. Furthermore, the outcome measures used in the clinical trials were often deemed inadequate in accurately capturing the complex nature of the conditions and the outcomes of interest to patients, thus falling short in comprehensively addressing the disease burden. Conversely, comments on the *economic evidence* and *costs* were raised only 8 times (1%).

Additionally, we analysed whether patient inputs consisted of novel insights or whether they were also raised by other stakeholders, namely manufacturers in the original submission or doctors and professional organisations in their testimonies, respectively (see Appendix B). Overall, we found that the majority of the themes raised by patients were also discussed by manufacturers (82%) in their application submission. Statements from doctors and professional groups were also overlapping in 45% of instances, mostly focused on clinically related themes, such as *unmet need*, *adverse events*, *clinical evidence*, and *symptoms*, with issues around *mental health* and *caregiver burden* almost never raised. Novel patients’ inputs (i.e., which were not discussed by other stakeholders) primarily focused on *access*, technology’s impact on *mental health*, and changes in *costs* for families and patients.

**Table 3:** Frequency of themes in patients’ inputs, by tier

Umbrella theme	*N* (%)	Tier 1 theme	*N* (%)	Tier 2 theme	*N* (%)
Disease-specific	345 (54%)	Treatment options	160 (25%)	Unmet need	59 (9%)
				Suboptimal treatment pathway	53 (8%)
				Access	48 (7%)
		Current quality of life	185 (29%)	Physical disabilities	58 (9%)
				Mental health	27 (4%)
				Daily life, social life, and education	42 (7%)
				Carer burden	58 (9%)
Technology-specific	237 (37%)	Quality of life with technology	148 (23%)	Independence	38 (6%)
				Symptoms	65 (10%)
				Mental health	29 (5%)
				Cost	16 (2%)
		Administration	60 (9%)	Frequency of administration	21 (3%)
				Ease of administration	39 (6%)
		Safety	29 (5%)	Adverse events	29 (5%)
Submission-specific	62 (10%)	Clinical evidence			54 (8%)
		Economic evidence			8 (1%)

*Note* The frequency is taken as the cumulative number of times each topic has occurred across the appraisals in scope. Percentages are calculated as the share among the overall number of unique patient inputs (*N* = 644)

### Influence of patients’ inputs in NICE final recommendations

Among the themes raised by patient organisations and experts in their written submissions, 48% were, on average, also explicitly mentioned in the FED, while the remaining (52%) were not (Table 4). Simply put, this means that for every patient input that was explicitly referenced to in NICE final decision document, there was roughly one that was not considered.

Overall, patients’ inputs explicitly mentioned in FEDs related to the disease (76%) rather than the technology being appraised (19%), with only 6% of submission-specific comments being discussed in both written submissions and FEDs. When looking more closely, the most frequent tier 1 patient inputs also explicitly discussed in the FEDs related to patients’ *current quality of life* aspects (53%), while those given least consideration were concerns around *administration* (1%). The tier 2 disease-specific themes that NICE committee members explicitly gave the highest consideration to were *daily life*, *social life and education*, which were present in all but one FED. This was closely followed by *carer burden*, *mental health*, and *physical disabilities*, which were both raised in most of the final recommendations. On the other hand, *mental health* (technology-specific) and *costs* issues were not explicitly considered in any of the FEDs, despite being raised in nine and seven of the written submissions, respectively. Finally, the themes of *independence* and *frequency of administration* have only been explicitly mentioned in two of the 15 appraisals, and *adverse events* were mentioned one time. Within submission-specific comments, patients mentioned *clinical* and *economic evidence* five and three times, respectively, in the appraisals analysed. However, patient comments on these topics were referenced nine and seven times, respectively, in the final NICE recommendations. While it may seem counterintuitive that the latter number is higher than the former, this is because patient comments on such topics are more likely to be raised during oral consultations after the discussion of manufacturer clinical and economic evidence, rather than in their initial written submissions, as analysed in this study.

Finally, when looking at individual appraisals, the share that themes mentioned in patients’ written submission explicitly considered in the FEDs ranged from 9 to 73% in the appraisals of onasemnogene abeparvovec (HST 24) and eladocagene exuparvovec (HST 26), respectively, with a median of 53%.

**Table 4:** Patient inputs explicitly considered in FEDs

**Umbrella theme**		**Tier 1 theme**		**Tier 2 theme**	**HST 17**			**HST 18**		**HST 19**		**HST 20**				**HST 21**			**HST 22**				**HST 23**			**HST 24**				**HST 25**				**HST 26**		
					**Odevixibat**			**Atidarsagene autotemcel**		**Elosulfase alfa**		**Selumetinib**				**Setmelanotide**			**Ataluren**				**Asfotase alfa**			**Onasemnogene abeparvovec**				**Lumasiran**				**Eladocagene exuparvovec**		
					**WS**	**FED**		**WS**	**FED**	**WS**	**FED**	**WS**		**FED**		**WS**	**FED**		**WS**		**FED**		**WS**	**FED**		**WS**		**FED**		**WS**		**FED**		**WS**	**FED**	
Disease-specific		Treatment options		Unmet need	✓	✓		✓	✓	✓	×	✓		×		✓	×		✓		×		✓	✓		✓		×		✓		✓		✓	✓	
				Suboptimal treatment pathway	✓	✓		✓	✓	✓	×	✓		×		✓	×		✓		×		✓	✓		✓		×		✓		✓		✓	×	
				Access	✓	×		✓	×	✓	✓	✓		✓		✓	×		✓		×		✓	×		✓		✓		✓		✓		✓	✓	
		Current quality of life		Physical disabilities	✓	✓		✓	✓	✓	×	✓		✓		✓	✓		✓		×		✓	✓		✓		×		✓		✓		✓	✓	
				Mental health	✓	✓		✓	✓	✓	×	✓		✓		✓	✓		✓		✓		✓	✓		×		×		✓		✓		✓	✓	
				Daily life, social life and education	✓	✓		✓	✓	✓	✓	✓		✓		✓	✓		✓		✓		✓	✓		✓		×		✓		✓		✓	✓	
				Carer burden	✓	✓		✓	✓	✓	×	✓		✓		✓	✓		✓		✓		✓	✓		✓		×		✓		✓		✓	✓	
Technology-specific		Quality of life with technology		Independence	✓	×		✓	×	✓	✓	✓		×		×	×		✓		×		✓	×		×		×		×		×		✓	×	
				Symptoms	✓	×		✓	×	✓	✓	✓		×		✓	✓		✓		✓		✓	✓		✓		×		✓		×		✓	✓	
				Mental health	×	×		✓	×	✓	×	✓		×		✓	×		✓		×		✓	×		×		×		×		×		×	×	
				Cost	×	×		✓	×	×	✓	✓		×		✓	×		✓		×		✓	×		×		×		×		×		×	×	
		Administration		Frequency of administration	×	×		✓	×	✓	✓	×		×		×	×		✓		×		✓	×		✓		×		✓		×		×	✓	
				Ease of administration	✓	×		✓	×	✓	✓	✓		×		✓	✓		✓		×		✓	×		×		×		✓		×		×	✓	
		Safety		Adverse events	×	×		✓	×	✓	×	×		×		✓	✓		✓		×		✓	×		✓		×		✓		×		✓	×	
Submission-specific		Clinical evidence			×	✓		×	✓	✓	✓	×		✓		×	✓		✓		✓		✓	×		✓		×		×		✓		✓	✓	
		Economic evidence			×	×		×	✓	✓	✓	×		×		×	✓		✓		✓		×	×		✓		×		×		✓		×	×	
Share (%) of themes mentioned WS explicitly considered in the FED					60%			43%		53%		42%				58%			38%				47%			9%				64%				73%		
**Umbrella theme**		**Tier 1 theme**				**Tier 2 theme**						**HST27**					**HST 28**				**HST 29**					**HST 30**				**HST 31**						
												**Afamelanotide**					**Birch bark extract**				**Velmanase alfa**					**Sebelipase alfa**				**Setmelanotide**						
												**WS**		**FED**			**WS**		**FED**		**WS**			**FED**		**WS**		**FED**		**WS**		**FED**				
Disease-specific		Treatment options				Unmet need						✓		✓			✓		✓		✓			×		✓		×		✓		×				
						Suboptimal treatment pathway						✓		✓			✓		✓		✓			×		✓		×		✓		×				
						Access						✓		×			✓		×		✓			✓		✓		✓		✓		×				
		Current quality of life				Physical disabilities						✓		✓			✓		✓		✓			×		✓		✓		✓		✓				
						Mental health						✓		✓			✓		✓		✓			×		✓		✓		✓		✓				
						Daily life, social life and education						✓		✓			✓		✓		✓			✓		✓		✓		✓		✓				
						Carer burden						✓		✓			✓		✓		✓			×		✓		✓		✓		✓				
Technology-specific		Quality of life with technology				Independence						✓		×			✓		×		✓			✓		✓		×		×		×				
						Symptoms						✓		×			✓		×		✓			✓		✓		×		✓		✓				
						Mental health						×		×			✓		×		✓			×		✓		×		✓		×				
						Cost						×		×			✓		×		×			✓		✓		×		✓		×				
		Administration				Frequency of administration						×		×			✓		×		✓			✓		×		×		×		×				
						Ease of administration						✓		×			✓		×		✓			✓		✓		×		✓		✓				
		Safety				Adverse events						×		×			✓		×		✓			×		×		×		✓		✓				
Submission-specific		Clinical evidence										×		✓			×		✓		✓			✓		×		✓		×		✓				
		Economic evidence										×		×			×		✓		✓			✓		×		×		×		✓				
Share (%) of themes mentioned WS explicitly considered in the FED												29%					60%				33%					64%				55%						

*Abbreviations* FED, Final Evaluation Document; HST, Highly Specialised Technology; WS, written submission

## Discussion

This study is the first to analyse and evaluate patient inputs to HST appraisals, and in the context of ultra-rare diseases. Our analysis finds that patient organisations and experts raise a wide range of themes in their inputs to NICE HST appraisals. Most of these pertain to disease-specific themes such as *carer burden*, *unmet need*, and *symptoms*, indicating that their testimonies are primarily based on their experiential accounts of either living with the condition or caring for someone with the condition. Patients’ inputs were found to overlap with statements from other stakeholders – particularly manufacturers – in the majority of the cases, with most novel inputs clustering around *access*, technology’s impact on *mental health*, and changes in *costs* for families and patients. Most of the contributing patient organisations reported funding from the manufacturer of the technology being appraised, ranging from £5,000 to £74,113. On average, about half of the themes raised in patients’ submission were referenced and explicitly attributed to patient organisations and experts in NICE final decisions, indicating that the HTA body is integrating patient inputs to some degree. However, the range is wide, with instances where issues important to patients do not correspond to being considered in the FED. Finally, the number of patient organisations and experts contributing to HST appraisals ranges between zero and three, with all experts being nominated by patient organisations and most organisations and expert contributing to only one appraisal.

Our findings align with the existing literature on the topic suggesting that patients contribute with experiential data pertaining how it is to live with a particular condition [6, 7, 26, 43, 57]. This is consistent with NICE’s decision modifiers, previously referred to as social value judgements, which are factors that NICE deems important but cannot be included in quality-adjusted life years (QALY) estimates. In fact, modifiers currently considered by NICE include the severity of the condition, which encompasses unmet medical need, and the size of benefit, which applies to HSTs exclusively [58]. This also resonates with the challenges in gathering economic and clinical evidence in ultra-rare diseases, which makes the role of patient inputs increasingly important in providing the full context of the disease being considered [6, 37, 38]. However, in our study, we do not find conclusive evidence that patient inputs hold more weight where uncertainty around clinical evidence is highest. For example, in the appraisal of elosulfase alfa, the committee stated that they were “disappointed that the company did not provide more robust analyses in its submission”, and we found that patient inputs are explicitly acknowledged in the FED 53% of the time. These findings might reflect the fact that, while patient inputs can provide clarity and insights into aspects of the disease, they may also introduce an additional element of uncertainty by adding more factors for decision-makers to consider. However, it is important to note that most of the technologies being appraised via the HST route have uncertain evidence and small clinical trials.

Concerns have been raised in the literature about potential conflicts of interests arising from patients’ involvement in HTA processes [12, 13]. First, our results suggest a lack a diversity of viewpoints. Specifically, our analysis found that a median of one patient organisation and two patient experts contributed to each appraisal. This limited representation may be attributed to the low prevalence and severity of the diseases assessed. For example, in afamelanotide’s appraisal (HST 27), the patient organisation providing input could not identify a patient expert to submit a statement due to the extremely rare nature of the disease under consideration. However, it raises questions about whether the experiences shared by contributing patients and organisations accurately reflect the broader population they aim to represent. Furthermore, certain patient organisations, such as The MPS Society, contributed to multiple HST appraisals. This may be due to the broad spectrum of diseases covered by these organisations and the challenges in recruiting groups focusing on specific diseases assessed. Nevertheless, it could also indicate that many smaller patient groups lack the necessary financial resources and expertise to participate effectively in these appraisals. This potential overrepresentation of a few but well-funded patient organisations echoes concerns raised by other scholars [6, 8, 25, 40].

Second, regarding the self-reported financial ties between patient organisations and the manufacturer of the technologies being appraised, such ties were found in 8 out of 15 appraisals, suggesting the presence of conflicts of interests. Most organisations reported funding only from the manufacturer of the appraised technology, with no disclosure of funding from competitors. This may be due to the ultra-rare nature of some diseases, where there are no direct competitors, but it could also be a result of misreporting or ambiguity regarding what qualifies as a competitor. We also observed inconsistent reporting of financial disclosures. In as many as one-third (5/15) of the organisational submissions, NICE did not request information on industry funding. Instead, patient organisations were asked if they had links with the tobacco industry. While this is relevant, it should be addressed as a separate question rather than an alternative, as this substitution results in inconsistent reporting. Finally, it remains unclear how NICE uses this information, including at what point these financial relationships can prevent participation in the HTA appraisal process, especially considering that the absolute value of funding is not informative without context regarding the patient organisation’s overall income.

Another issue rarely discussed in the literature, but routinely faced by HTA bodies, is the opportunity cost associated with the reimbursement of certain technologies and the impact this has on other patient groups with conditions not under consideration. NICE has attempted to address this issue by involving the public, including those affected by these opportunity costs, through a Citizens’ Council, which has now been replaced by a public engagement initiative called NICE Listens. However, it remains unclear whether these initiatives have an impact on future decisions and whether NICE has the resources to ensure meaningful and sustained public involvement [59].

Finally, in the evidence analysed, patients raised concerns about the limited use of real-world evidence compared to clinical trial data. They also criticised some of the outcome measures used in trials, deeming them inadequate in capturing the complex nature of the diseases under assessment. While it is important for HTA bodies to adapt to the changes in clinical development and keep up with the use of real-world-evidence and of surrogate endpoints, when necessary, they must maintain a clear stance in balancing patients’ right to access promising medicines without compromising clinical standards in the name of flexibility.

Applying the 3I’s framework helps us make sense of the results discussed above. For example, the overlap found between patient and manufacturer inputs, suggests that patients’ voices (and their *interests* as defined in the conceptual framework) are aligned with industry as they wish to gain faster access to technologies being appraised. However, this result should be interpreted with caution, as the overlap might be spurious and could be attributable to the comprehensiveness of manufacturers’ submissions. Turning to the *ideas* dimension, NICE’s integration of patient themes suggest that its utilitarian approach favours quantifiable outcomes that suit economic evaluations. While issues such as severity are explicitly addressed in NICE decision modifiers, qualitative aspects like mental health and carer burden receive limited attention. Patients, however, often prioritise personal experience over statistical precision, highlighting how economic models may overlook factors that affect their lives. This suggests NICE’s model might benefit from added flexibility to better capture non-clinical impacts. Finally, from an *institutional* perspective, NICE’s framework for patient engagement tends to limit the diversity of patient contributions to a few organizations, potentially unrepresentative of the broader population affected by its decisions. Additionally, inconsistencies in the disclosure of pharmaceutical funding may limit NICE’s ability to identify conflicts of interest, which, if unaddressed, could undermine the credibility of patient inputs and diminish their impact on final recommendations intended to benefit patients.

The study presented here should be viewed in light of its limitations. First, the study considers 15 of the most recent HST appraisals and therefore might not be representative of all HST appraisals. Second, this study has considered the initial written submissions only from the first committee meeting. This approach was chosen for the sake of comparability across different appraisals and their associated documents; however, this probably led to some patient inputs being overlooked. Third, our methodology only accounted for explicit references to patient testimonies in NICE final recommendations. As a result, situations where patients’ inputs indirectly influenced the committee’s final decision or raised issues also discussed by other stakeholders, such as doctors, were not considered in our assessment.

Despite these caveats, the findings from our study advance our understanding on patient inputs to NICE appraisals and how they are considered in final recommendations. Furthermore, we contribute to the existing literature by expanding the conceptual understanding of the dynamics between patient organisations, experts, NICE, and manufacturers during the reimbursement decision-making process, as well as deploying an underused but high-potential methodology in the field, namely document analysis. Policymakers should consider these results when planning whether and how to gauge patients’ inputs in HTA. Specifically, HTA bodies might revise their existing guidelines on patient involvement and consider implementing an impact assessment to ensure their efforts in capturing patients’ experiences align with their intended objectives and are not merely tokenistic. Additionally, to improve patient representation and increase the trust of committee members in the inputs from patient organisations and experts, policymakers should adopt tools to ensure the incorporation of a variety of viewpoints and reduce the risk of bias from pharmaceutical companies funding patient groups, while also ensuring adequate representation of patient perspectives.

Building on this study, future research could delve deeper into patient inputs for a specific HST appraisal, conducting a case study analysis involving a wider range of documents. This approach might allow a more profound understanding of whether patient inputs can influence the reimbursement decision, rather than providing information that, while acknowledged in NICE’s final decision, primarily enhances the committee’s understanding of the condition or technology being appraised. Moreover, this analysis could be integrated with older and/or new submissions, providing a more comprehensive view of the evolution of patient involvement. Subsequent studies could investigate the financial reliance of patient representative organisations on funding from manufacturers through external sources and compare these values with those declared in NICE forms. Additionally, they could assess whether and how such financial ties might bias committee members against the organisational submissions. Finally, this study design might be replicated across different HTA bodies and jurisdictions to allow international comparisons and highlight implicit value judgments of committee members and how different systems integrate patients’ voices into their appraisals.

## Conclusions

Over the past decades, HTA bodies have taken significant steps to integrate or consolidate patients’ inputs into their processes. The findings of this study highlight that patients primarily contribute their experiences of living with diseases, offering novel insights into areas such as the burden on caregivers and the impact of the disease on their mental health. For each theme raised by patients and explicitly acknowledged in NICE committee’s final recommendations, one is not, indicating room for improvement in NICE’s consideration of patient inputs. Additionally, financial ties between patient organisations and manufacturers were disclosed in the majority of appraisals, raising questions about potential industry influence and highlighting the need for greater transparency and mitigation strategies. More research is needed to examine when and in which areas patient contributions can be more useful, whether diversity of viewpoints is accounted for, and how NICE and other HTA bodies can streamline their involvement.

## Electronic supplementary material

Below is the link to the electronic supplementary material.

Supplementary Material 1

## Data Availability

All data used in this article are publicly available. Links to the data sources are provided in the Appendix.

## Appendix

A. ***Patient organisations’ involvement into NICE process***.

As part of its deliberative process, NICE involves all key stakeholders in its decision process, including patients and clinical experts. During this process, patient organisations are invited to provide submissions, respond to consultations, and nominate patient experts to participate in the process [54, 60, 61]. Specifically, the NICE technology appraisal process is divided into two stages: scoping and guidance development. During the scoping stage NICE decides which technologies to assess, and patient organisations can provide inputs through a written consultation (scoping consultation) and an oral consultation (scoping workshop) [61]. The scoping consultation allows groups to provide comments on the draft scope and remit of the appraisal, while the scoping workshop provides an opportunity to participate in discussions after written comments are received. During the guidance development stage, patient organisations can provide a written submission to highlight their views on the technology being appraised, which is reviewed alongside clinical and economic evidence submitted from the manufacturer, clinical experts and other stakeholders [54]. Patient organisations are selected to participate in the process through two methods: registering interest on the NICE website or being invited by NICE. NICE reaches out to previous stakeholders or those potentially interested in the topic [54]. Contributors may receive up to £400 in financial compensation [54].

Patient organisations can also nominate patient experts to attend part of the appraisal committee meeting and provide written submissions as individuals, not as representatives of their nominating organisation. Typically, two patient experts are nominated per appraisal, one with broad knowledge of the condition, treatments, and outcomes important to patients, and one with personal experience of the condition and treatment, if possible [62]. However, if no patient organisation takes part to the appraisal, patient expert might not be nominated and, therefore, are recruited independently.

Organisational and patient inputs follow a predetermined structure and present consultees with questions around the following domains: living with the condition, current treatment of the condition in the NHS, advantages of the technology, disadvantages of the technology, patient population, equality and other issues [63]. Finally, the technical team can add topic-specific questions if needed.

B. ***Novelty of patient inputs***.

**Table:** Comparison of patient inputs with statements from other stakeholders

Umbrella theme	Tier 1 theme	Tier 2 theme	HST 17			HST 18			HST 19			HST 20			HST 21			HST 22		
			PO	M	D/PG	PO	M	D/PG	PO	M	D/PG	PO	M	D/PG	PO	M	D/PG	PO	M	D/PG
**Disease-specific**	Treatment options	Unmet need	✓	✓	✓	✓	✓	✓	✓	✓	✓	✓	✓	✓	✓	✓	✓	✓	✓	✓
		Suboptimal treatment pathway	✓	✓	✓	✓	✓	✓	✓	×	×	✓	✓	✓	✓	✓	✓	✓	×	×
		Access	✓	×	×	✓	✓	×	✓	×	✓	✓	×	✓	✓	×	×	✓	×	✓
	Current quality of life	Physical disabilities	✓	✓	✓	✓	✓	×	✓	✓	✓	✓	✓	✓	✓	✓	✓	✓	✓	×
		Mental health	✓	✓	×	✓	✓	×	✓	✓	×	✓	✓	×	✓	✓	×	✓	×	×
		Daily life, social life and education	✓	✓	×	✓	✓	×	✓	✓	×	✓	✓	✓	✓	✓	✓	✓	✓	×
		Carer burden	✓	✓	×	✓	✓	×	✓	✓	×	✓	✓	×	✓	✓	×	✓	✓	×
**Technology-specific**	Quality of life with technology	Independence	✓	×	×	✓	✓	×	✓	✓	✓	✓	✓	×	×	-	-	✓	✓	✓
		Symptoms	✓	✓	✓	✓	✓	×	✓	✓	✓	✓	✓	✓	✓	✓	✓	✓	✓	✓
		Mental health	×	-	-	✓	✓	×	✓	✓	×	✓	✓	×	✓	×	×	✓	×	×
		Cost	×	-	-	✓	✓	×	×	-	-	✓	✓	×	✓	×	×	✓	✓	×
	Administration	Frequency of administration	×	-	-	✓	✓	✓	✓	×	×	×	-	-	×	-	-	✓	×	✓
		Ease of administration	✓	×	×	✓	✓	✓	✓	×	✓	✓	✓	×	✓	×	✓	✓	×	✓
	Safety	Adverse events	×	-	-	✓	✓	✓	✓	✓	✓	×	-	-	✓	✓	✓	✓	✓	✓
**Submission-specific**	Clinical evidence		×	-	-	×	-	-	✓	✓	✓	×	-	-	×	-	-	✓	✓	✓
	Economic evidence		×	-	-	×	-	-	✓	✓	×	×	-	-	×	-	-	✓	✓	×
Umbrella theme	Tier 1 theme	Tier 2 theme	HST 23			HST 24			HST 25			HST 26			HST 27			HST 28		
			PO	M	D/PG	PO	M	D/PG	PO	M	D/PG	PO	M	D/PG	PO	M	D/PG	PO	M	D/PG
**Disease-specific**	Treatment options	Unmet need	✓	✓	×	✓	✓	×	✓	✓	✓	✓	✓	✓	✓	✓	✓	✓	✓	✓
		Suboptimal treatment pathway	✓	×	×	✓	✓	✓	✓	✓	✓	✓	✓	×	✓	✓	×	✓	×	×
		Access	✓	✓	×	✓	×	✓	✓	✓	✓	✓	×	×	✓	×	✓	✓	×	×
	Current quality of life	Physical disabilities	✓	✓	×	✓	✓	×	✓	✓	✓	✓	✓	×	✓	✓	×	✓	✓	✓
		Mental health	✓	✓	×	×	-	-	✓	✓	×	✓	✓	×	✓	✓	×	✓	✓	×
		Daily life, social life and education	✓	✓	×	✓	✓	×	✓	✓	×	✓	✓	×	✓	✓	×	✓	✓	×
		Carer burden	✓	✓	×	✓	✓	✓	✓	✓	×	✓	✓	×	✓	✓	×	✓	✓	×
**Technology-specific**	Quality of life with technology	Independence	✓	×	×	×	-	-	×	-	-	✓	✓	×	✓	✓	×	✓	✓	×
		Symptoms	✓	✓	×	✓	✓	✓	✓	✓	✓	✓	✓	×	✓	✓	✓	✓	✓	✓
		Mental health	✓	×	×	×	-	-	×	-	-	×	-	-	✓	✓	×	×	-	-
		Cost	✓	×	×	×	-	-	×	-	-	×	-	-	✓	×	×	×	-	-
	Administration	Frequency of administration	✓	×	×	✓	✓	✓	✓	✓	✓	×	-	-	✓	✓	✓	×	-	-
		Ease of administration	✓	×	×	×	-	-	✓	×	✓	×	-	-	✓	✓	✓	×	-	-
	Safety	Adverse events	✓	✓	×	✓	✓	✓	✓	✓	✓	✓	✓	✓	✓	✓	✓	✓	✓	✓
**Submission-specific**	Clinical evidence		✓	✓	×	✓	✓	✓	×	-	-	✓	✓	✓		×	-	-	×	-
	Economic evidence		×	-	-	✓	✓	×	×	-	-	×	-	-		×	-	-	×	-
Umbrella theme	Tier 1 theme	Tier 2 theme	HST 29			HST 30			HST 31											
			PO	M	D/PG	PO	M	D/PG	PO	M	D/PG									
**Disease-specific**	Treatment options	Unmet need	✓	✓	✓	✓	✓	✓	✓	✓	✓									
		Suboptimal treatment pathway	✓	✓	✓	✓	✓	×	✓	✓	✓									
		Access	✓	✓	✓	✓	✓	✓	✓	✓	✓									
	Current quality of life	Physical disabilities	✓	✓	✓	✓	✓	✓	✓	✓	✓									
		Mental health	×	-	-	×	-	-	✓	✓	×									
		Daily life, social life and education	✓	✓	×	✓	✓	×	✓	✓	✓									
		Carer burden	✓	✓	×	✓	✓	×	✓	✓	✓									
**Technology-specific**	Quality of life with technology	Independence	✓	✓	×	✓	×	×	×	-	-									
		Symptoms	✓	✓	✓	✓	✓	×	✓	✓	✓									
		Mental health	✓	×	×	×	-	-	✓	✓	×									
		Cost	✓	×	×	×	-	-	×	-	-									
	Administration	Frequency of administration	×	-	-	✓	✓	×	✓	✓	×									
		Ease of administration	✓	✓	✓	✓	✓	×	✓	✓	✓									
	Safety	Adverse events	✓	✓	✓	✓	✓	×	×	-	-									
**Submission-specific**	Clinical evidence		✓	×	-	-	×	-	-	×	-									
	Economic evidence		×	×	-	-	×	-	-	×	-									

Abbreviations: D, Doctors; M, Manufacturers; PG, Professional groups; PO, Patient organisations

C. ***Data sources***.

**Table:**

HST	NICE (Committee papers)	NICE (FED or FDG)	G-BA ratings	HAS ratings (SMR, ASMR)
**17**	https://www.nice.org.uk/guidance/hst17/documents/committee-papers	https://www.nice.org.uk/guidance/hst17/documents/final-evaluation-determination-document	https://www.g-ba.de/downloads/39-1464-5313/2022-03-03_AM-RL-XII_Odevixibat_D-725_EN.pdf	https://www.has-sante.fr/jcms/p_3299696/fr/bylvay-odevixibat#smr
**18**	https://www.nice.org.uk/guidance/hst18/documents/committee-papers	https://www.nice.org.uk/guidance/hst18/documents/final-evaluation-determination-document-3	https://www.g-ba.de/downloads/40-1465-7994/2021-11-04_AM-RL-XII_Atidarsagen-autotemcel_D-678_TrG_EN.pdf	https://www.has-sante.fr/jcms/p_3263243/fr/libmeldy-population-autologue-enrichie-en-cellules-cd34-qui-contient-des-cellules-souches-progenitrices-hematopoietiques-transduites-ex-vivo-avec-un-vecteur-lentiviral-codant-le-gene-de-l-arylsulfatase-a-humaine
**19**	https://www.nice.org.uk/guidance/hst19/documents/committee-papers	https://www.nice.org.uk/guidance/hst19/documents/final-evaluation-determination-document	https://www.g-ba.de/downloads/92-975-2085/2017-09-15_Nutzenbewertung_G-BA_Elosulfase-alfa_D-320.pdf	https://www.has-sante.fr/jcms/p_3448570/fr/vimizim-elosulfase-alfa-mucopolysaccharidose-de-type-iv-a-syndrome-de-morquio-a
**20**	https://www.nice.org.uk/guidance/hst20/documents/committee-papers	https://www.nice.org.uk/guidance/hst20/documents/final-evaluation-determination-document	https://www.g-ba.de/downloads/40-1465-8235/2022-02-03_AM-RL-XII_Selumetinib_D-714_TrG_EN.pdf	https://www.has-sante.fr/jcms/p_3322781/fr/koselugo-selumetinib-neurofibromes-plexiformes
**21**	https://www.nice.org.uk/guidance/hst21/documents/committee-papers	https://www.nice.org.uk/guidance/hst21/documents/final-evaluation-determination-document	https://www.g-ba.de/downloads/40-1465-9078/2022-12-01_AM-RL-XII_Setmelanotide_D-824_TrG_EN.pdf	https://www.has-sante.fr/jcms/p_3416571/fr/imcivree-setmelanotide-pomc
**22**	https://www.nice.org.uk/guidance/hst22/documents/committee-papers	https://www.nice.org.uk/guidance/hst22/documents/final-evaluation-determination-document-2	https://www.g-ba.de/downloads/40-1465-4072/2016-12-01_AM-RL-XII_Ataluren_D-239_TrG_EN.pdf	https://www.has-sante.fr/jcms/p_3118134/fr/translarna-ataluren
**23**	https://www.nice.org.uk/guidance/hst23/documents/committee-papers	https://www.nice.org.uk/guidance/hst23/documents/final-evaluation-determination-document	https://www.g-ba.de/downloads/40-1465-3662/2016-03-17_AM-RL-XII_Asfotase-alfa_D-188_TrG_EN.pdf	https://www.has-sante.fr/jcms/p_3455973/fr/strensiq-asfotase-alfa-hypophosphatasie
**24**	https://www.nice.org.uk/guidance/hst24/documents/committee-papers	https://www.nice.org.uk/guidance/hst24/documents/final-evaluation-determination-document	https://www.g-ba.de/bewertungsverfahren/nutzenbewertung/689/#beschluesse	https://www.has-sante.fr/jcms/p_3442932/fr/zolgensma-onasemnogene-abeparvovec-amyotrophie-spinale
**25**	https://www.nice.org.uk/guidance/hst25/documents/committee-papers	https://www.nice.org.uk/guidance/hst25/documents/final-appraisal-determination-document	https://www.g-ba.de/downloads/40-1465-7656/2021-07-01_AM-RL-XII_Lumasiran_D-622_TrG_EN.pdf	https://www.has-sante.fr/jcms/p_3266578/fr/oxlumo-94-5-mg/0-5-ml-lumasiran
**26**	https://www.nice.org.uk/guidance/hst26/documents/committee-papers	https://www.nice.org.uk/guidance/hst26/documents/final-evaluation-determination-document	https://www.g-ba.de/downloads/40-1465-9247/2023-02-02_AM-RL-XII_Eladocagene-Exuparvovec_D-856_TrG_EN.pdf	https://www.has-sante.fr/jcms/p_3402378/fr/upstaza-eladocagene-exuparvovec-deficit-en-decarboxylase-d-acide-l-amine-aromatique
**27**	https://www.nice.org.uk/guidance/hst27/documents/committee-papers	https://www.nice.org.uk/guidance/hst27/documents/final-evaluation-determination-document	https://www.g-ba.de/downloads/40-1465-7652/2021-07-01_AM-RL-XII_Afamelanotid_D-641_TrG_EN.pdf	https://www.has-sante.fr/jcms/p_3192396/fr/scenesse-afamelanotide
**28**	https://www.nice.org.uk/guidance/hst28/documents/committee-papers	https://www.nice.org.uk/guidance/hst28/documents/674	https://www.g-ba.de/downloads/40-1465-9274/2023-02-16_AM-RL-XII_Birch-Bark-Extract_D-862_TrG_EN.pdf	https://www.has-sante.fr/jcms/p_3394096/fr/filsuvez-extrait-sec-raffine-d-ecorce-de-bouleau-epidermolyse-bulleuse-dystrophique
**29**	https://www.nice.org.uk/guidance/hst29/documents/committee-papers	https://www.nice.org.uk/guidance/hst29/documents/final-evaluation-determination-document	https://www.g-ba.de/downloads/39-261-3617/2018-12-20_AM-RL-XII_Velmanase-alfa_D-365_BAnz.pdf	https://www.has-sante.fr/jcms/p_3313547/fr/lamzede-velmanase-alfa-alpha-mannosidose-legere-a-moderee
**30**	https://www.nice.org.uk/guidance/hst30/documents/committee-papers	https://www.nice.org.uk/guidance/hst30/documents/674	https://www.g-ba.de/downloads/40-1465-7570/2021-06-03_AM-RL-XII_Sebelipase-alfa_D-606_TrG_EN.pdf	
**31**	https://www.nice.org.uk/guidance/hst31/documents/committee-papers	https://www.nice.org.uk/guidance/hst31/documents/674	https://www.g-ba.de/downloads/40-1465-9934/2023-11-02_AM-RL-XII_Setmelanotide_D-941_TrG_EN.pdf	https://www.has-sante.fr/jcms/p_3421149/fr/imcivree-setmelanotide-syndrome-de-bardet-biedl-sbb

## References

[1] Richard, L.K., Joy, M.: Engaging patients in medical decision making. BMJ. **323**(7313), 584 (2001). 10.1136/bmj.323.7313.584 11557690 10.1136/bmj.323.7313.584 PMC1121170

[2] Cumberlege, J.: First Do no Harm - The Report of the Independent Medicines and Medical Devices Safety Review. In. The Independent Medicines and Medical Devices Safety Review (2020) 10.1177/00369330211058467 34994662

[3] Haskell, H.: Cumberlege review exposes stubborn and dangerous flaws in healthcare. BMJ. **370**(m3099) (2020). 10.1136/bmj.m3099 10.1136/bmj.m3099 32763955

[4] Timpe, C., Stegemann, S., Barrett, A., Mujumdar, S.: Challenges and opportunities to include patient-centric product design in industrial medicines development to improve therapeutic goals. Br. J. Clin. Pharmacol. **86**(10), 2020–2027 (2020). 10.1111/bcp.14388 32441052 10.1111/bcp.14388 PMC7495299

[5] Campbell, B., Sedrakyan, A.: Patient involvement in regulation: An unvalued imperative. Lancet. **397**(10290), 2147–2148 (2021). 10.1016/S0140-6736(21)00977-6 34090599 10.1016/S0140-6736(21)00977-6

[6] Facey, K.: As health technology assessment evolves so must its approach to patient involvement. J. Comp. Eff. Res. **8**(8), 549–554 (2019). 10.2217/cer-2019-0039 31116026 10.2217/cer-2019-0039

[7] Weeks, L., Polisena, J., Scott, A.M., Holtorf, A.-P., Staniszewska, S., Facey, K.: Evaluation of patient and public involvement initiatives in health technology assessment: A survey of international agencies. Int. J. Technol. Assess. Health Care. **33**(6), 715–723 (2017). 10.1017/S0266462317000976 29122048 10.1017/S0266462317000976

[8] Gesbert, C., André-Vert, J., Guerrier, M., Galbraith, M., Devaud, C., Dupont, J.-C.K., Mamzer, M.-F.: The contribution of French patient and consumer groups to health technology assessments over a 2-year period: An observational retrospective study. Int. J. Technol. Assess. Health Care. **37**(1) (2021). 10.1017/S0266462321000180 e48 10.1017/S0266462321000180 33745474

[9] Livingstone, H., Verdiel, V., Crosbie, H., Upadhyaya, S., Harris, K., Thomas, L.: Evaluation of the impact of patient input in health technology assessments at NICE. (1471–6348 (2021). (Electronic)) 10.1017/S0266462320002214 33509314

[10] Edwards, K.T.: The role of patient participation in Drug approvals lessons from the Accelerated approval of Eteplirsen. Food Drug Law J. **72**(3), 406–450 (2017)

[11] Gentilini, A.P.I.: Industry funding of patient organisations in the UK: a retrospective study of commercial determinants, funding concentration and disease prevalence. BMJ open 13(6), e071138 (2023). 10.1136/bmjopen-2022-071138, chapter = 0 10.1136/bmjopen-2022-071138 PMC10410975 37369404

[12] Parvanova, I., Gentilini, A., Cushing, J., Naci, H.: Safeguarding NICE from patient groups’ conflicts of interest. BMJ. **381**, 1243 (2023). 10.1136/bmj.p1243 37257888 10.1136/bmj.p1243

[13] Mandeville, K.L., Barker, R., Packham, A., Sowerby, C., Yarrow, K., Patrick, H.: Financial interests of patient organisations contributing to technology assessment at England’s National Institute for Health and Care Excellence: Policy review. BMJ. **364**, k5300 (2019). 10.1136/bmj.k5300 30651227 10.1136/bmj.k5300 PMC6334181

[14] Lynch, H.F., Largent, E.A.: Considering tomorrow’s patients in today’s drug approvals. BMJ. **381** (2023). 10.1136/bmj-2023-075000 e075000 10.1136/bmj-2023-075000 37290781

[15] Tonkinson, A., Livingstone, H., Upadhyaya, S., Leng, G.: OP21 enhancing capability: Patient impact in Ultra-orphan conditions. Int. J. Technol. Assess. Health Care. **35**(S1), 4–4 (2019). 10.1017/S026646231900093X

[16] Smit, C.: Personal reflections of a Patient representative in an Appraisal Committee. Patient. **8** (2014). 10.1007/s40271-014-0086-8 10.1007/s40271-014-0086-8 25256116

[17] Menon, D., Stafinski, T., Dunn, A., Short, H.: Involving patients in reducing decision uncertainties around orphan and ultra-orphan drugs: A rare opportunity? Patient-Patient-Centered Outcomes Res. **8**, 29–39 (2015) 10.1007/s40271-014-0106-8 25516506

[18] Smith, C.I.E., Bergman, P., Hagey, D.W.: Estimating the number of diseases - the concept of rare, ultra-rare, and hyper-rare. (2022). (2589-0042 (Electronic)) 10.1016/j.isci.2022.104698 PMC9287598 35856030

[19] Whittal, A.A.-O., Meregaglia, M.A.-O., Nicod, E.A.-O.: The Use of Patient-Reported Outcome Measures in Rare Diseases and Implications for Health Technology Assessment. (1178–1661 (Electronic)) (2021) 10.1007/s40271-020-00493-w PMC8357707 33462774

[20] Meregaglia, M., Nicod, E., Drummond, M.: The estimation of health state utility values in rare diseases: Do the approaches in submissions for NICE technology appraisals reflect the existing literature? A scoping review. Eur. J. Health Econ. (2022). 10.1007/s10198-022-01541-y 10.1007/s10198-022-01541-y PMC10406664 36335234

[21] Nicod, E., Berg Brigham, K., Durand-Zaleski, I., Kanavos, P.: Dealing with uncertainty and accounting for Social Value judgments in assessments of Orphan drugs: Evidence from four European countries. (1524–4733 (2017). (Electronic)) 10.1016/j.jval.2017.03.005 28712621

[22] Nicod, E., Annemans, L., Bucsics, A., Lee, A., Upadhyaya, S., Facey, K.: HTA programme response to the challenges of dealing with orphan medicinal products: Process evaluation in selected European countries. (1872–6054 (Electronic)) (2019) 10.1016/j.healthpol.2017.03.009 28400128

[23] Nicod, E., Kanavos, P.: Scientific and Social Value Judgements for Orphan Drugs in Health Technology Assessment. (1471–6348 (Electronic)) (2016) 10.1017/S0266462316000416 27624559

[24] NICE: Interim Process and Methods of the Highly Specialised Technologies Programme: (Updated to reflect 2017 changes 2017). (2023). https://www.nice.org.uk/Media/Default/About/what-we-do/NICE-guidance/NICE-highly-specialised-technologies-guidance/HST-interim-methods-process-guide-may-17.pdf. 2023 27905709

[25] Mercer, R.E., Chambers, A., Mai, H., McDonald, V., McMahon, C., Chan, K.K.W.: Are we making a difference? A qualitative study of Patient Engagement at the pan-canadian Oncology Drug Review: Perspectives of patient groups. (2020). (1524–4733 (Electronic)) 10.1016/j.jval.2020.06.003 32940233

[26] Staley, K., Doherty, C.: It’s not evidence, it’s insight: Bringing patients’ perspectives into health technology appraisal at NICE. Res. Involv. Engagem. **2**(1), 4 (2016). 10.1186/s40900-016-0018-y 29062505 10.1186/s40900-016-0018-y PMC5611625

[27] Dipankui, M.T., Gagnon, M.P., Desmartis, M., Légaré, F., Piron, F., Gagnon, J., Rhiands, M., Coulombe, M.: Evaluation of patient involvement in a health technology assessment. Int. J. Technol. Assess. Health Care. **31**(3), 166–170 (2015). 10.1017/s0266462315000240 26062904 10.1017/S0266462315000240

[28] Chang, R., Versoza, L., Jaksa, A., Ho, Y.: PSY82 - how influential are patient and professional group submissions on reimbursement decisions for European Medicines Agency Orphan drugs? Value Health. **18**(3), A306 (2015). 10.1016/j.jval.2015.03.1780

[29] Hamilton, K.A., Griffiths, M., Hanman, K.: Patient Group submissions (PGSS) in Health Technology Assessment (HTA) in Scotland: Prevalence and Impact. Value Health. **19**(7), A440 (2016). 10.1016/j.jval.2016.09.540

[30] Smith, N., Mitton, C., Davidson, A., Williams, I.: A politics of priority setting: Ideas, interests and institutions in healthcare resource allocation. Public. Policy Adm. **29**(4), 331–347 (2014). 10.1177/0952076714529141

[31] Norburn, L.A.-O., Thomas, L.A.-O.: Expertise, experience, and excellence. Twenty years of patient involvement in health technology assessment at NICE: an evolving story. (1471–6348 (Electronic)) (2020) 10.1017/S0266462320000860 33168114

[32] Angelis, A., Lange, A., Kanavos, P.: Using health technology assessment to assess the value of new medicines: Results of a systematic review and expert consultation across eight European countries. Eur. J. Health Econ. **19**(1), 123–152 (2018). 10.1007/s10198-017-0871-0 28303438 10.1007/s10198-017-0871-0 PMC5773640

[33] House of Commons - Health Committee: The Influence of the Pharmaceutical Industry. In: Fourth Report of Session 2004–05: (2005)

[34] Office for National Statistics: Population and household estimates, England and Wales: Census 2021, unrounded data. (2022). https://www.ons.gov.uk/peoplepopulationandcommunity/populationandmigration/populationestimates/bulletins/populationandhouseholdestimatesenglandandwales/census2021unroundeddata

[35] NICE: Technology appraisal guidance - Technology appraisals and the NHS Constitution. (2023). https://www.nice.org.uk/about/what-we-do/our-programmes/nice-guidance/nice-technology-appraisal-guidance#:~:text=The%20NHS%20is%20legally%20obliged,believes%20they%20are%20clinically%20appropriate

[36] Facey Karen, M.: As health technology assessment evolves so must its approach to patient involvement. J. Comp. Eff. Res. **8**(8), 549–554 (2019). 10.2217/cer-2019-0039 31116026 10.2217/cer-2019-0039

[37] Wale, J., Scott, A.M., Hofmann, B., Garner, S., Low, E., Sansom, L.: WHY PATIENTS SHOULD BE INVOLVED IN HEALTH TECHNOLOGY ASSESSMENT. (1471–6348 (Electronic)) (2017) 10.1017/S0266462317000241 28528585

[38] Boothe, K.: (re)defining legitimacy in Canadian drug assessment policy? Comparing ideas over time. Health Econ. Policy Law. **16**(4), 424–439 (2021). 10.1017/s1744133121000013 33557999 10.1017/S1744133121000013 PMC8460446

[39] Lexchin, J.: Association between commercial funding of Canadian patient groups and their views about funding of medicines: An observational study. PloS One. **14**(2), e0212399 (2019). 10.1371/journal.pone.0212399 30768629 10.1371/journal.pone.0212399 PMC6377138

[40] Gagnon, M.P., Desmartis, M., Fau - Lepage-Savary, D., Lepage-Savary, D., Fau - Gagnon, J., Gagnon, J., Fau - St-Pierre, M., St-Pierre, M., Fau - Rhainds, M., Rhainds, M.F., Lemieux, R., Lemieux, R.F., Gauvin, F.-P.: Gauvin Fp Fau - Pollender, H., Pollender H Fau - Légaré, F., Légaré, F.: Introducing patients’ and the public’s perspectives to health technology assessment: A systematic review of international experiences. (1471–6348 (Electronic)) (2011) 10.1017/S0266462310001315 21262085

[41] Gentilini, A., Parvanova, I.: Industry funding of patient organisations in the UK: A retrospective study of commercial determinants, funding concentration and disease prevalence. BMJ Open. **13**(6), e071138 (2023). 10.1136/bmjopen-2022-071138 10.1136/bmjopen-2022-071138 PMC10410975 37369404

[42] Barnes, M., Newman, J., Sullivan, H.: Power, participation and political renewal: Theoretical and empirical perspectives on public participation under New Labour. Social Politics. **11**, 267–279 (2004)

[43] Menon, D., Stafinski, T.: Role of patient and public participation in health technology assessment and coverage decisions. (1744–8379 (Electronic)) (2014) 10.1586/erp.10.82 21351860

[44] Cavazza, M., Jommi, C.: Stakeholders involvement by HTA organisations: Why is so different? Health Policy. **105**(2), 236–245 (2012). 10.1016/j.healthpol.2012.01.012 22364715 10.1016/j.healthpol.2012.01.012

[45] Scott, A.M., Wale, J.L.: On behalf of the Htai Patient and Citizen Involvement in Hta Interest Group, P.I.a.E.W.G.: Patient advocate perspectives on involvement in HTA: an international snapshot. Research Involvement and Engagement 3(1), 2 (2017). 10.1186/s40900-016-0052-9 10.1186/s40900-016-0053-8 PMC5611570 29062528

[46] de Corte: K.a.C.J.a.G.R.: Stated versus revealed preferences: An approach to reduce bias. Health Econ. **30**(5), 1095–1123 (2021). 10.1002/hec.4246 10.1002/hec.4246 33690931

[47] Johansson-Stenman, O., Svedsäter, H.: Self-image and valuation of moral goods: Stated versus actual willingness to pay. J. Econ. Behav. Organ. **84**(3), 879–891 (2012). 10.1016/j.jebo.2012.10.006

[48] Taylor, S.E., Brown, J.D.: Positive illusions and well-being revisited: Separating fact from fiction. Psychol. Bull. **116**(1), 21–27 (1994). 10.1037/0033-2909.116.1.21 discussion 28 8078971 10.1037/0033-2909.116.1.21

[49] Cockerill, K., Gaebler, J.A., HT5 - FINDINGS FROM THE FIRST FIVE YEARS OF THE UK NICE HST PROGRAM: Value Health. **21** (2018). 10.1016/j.jval.2018.09.051

[50] Angelis, A., Harker, M., Cairns, J., Seo, M.K., Legood, R., Miners, A., Wiseman, V., Chalkidou, K., Grieve, R., Briggs, A.: The evolving nature of Health Technology Assessment: A critical Appraisal of NICE’s New methods Manual. Value Health. **26**(10), 1503–1509 (2023). 10.1016/j.jval.2023.05.015 37268059 10.1016/j.jval.2023.05.015

[51] Berglas, S., Jutai, L., MacKean, G., Weeks, L.: Patients’ perspectives can be integrated in health technology assessments: An exploratory analysis of CADTH Common Drug Review. Res. Involv. Engagem. **2**(1), 21 (2016). 10.1186/s40900-016-0036-9 29062521 10.1186/s40900-016-0036-9 PMC5611639

[52] Barlow, P., Gleeson, D., O’Brien, P., Labonte, R.: Industry influence over global alcohol policies via the World Trade Organization: A qualitative analysis of discussions on alcohol health warning labelling, 2010–19. Lancet Global Health. **10**(3), e429–e437 (2022). 10.1016/S2214-109X(21)00570-2 35120586 10.1016/S2214-109X(21)00570-2

[53] Ozieranski, P., Rickard, E., Mulinari, S.: Exposing drug industry funding of UK patient organisations. BMJ. **365**, l1806 (2019). 10.1136/bmj.l1806 31122928 10.1136/bmj.l1806 PMC6529850

[54] NICE: Public Involvement Programme - Developing technology appraisals: a factsheet for patient and carer groups https:// (2014). www.nice.org.uk/Media/Default/About/NICE-Communities/Public-involvement/Developing-NICE-guidance/Developing-technology-appraisals-factsheet-patient-carer-groups.pdf

[55] La Vaque, T.J., Rossiter, T.: The ethical use of Placebo controls in Clinical Research: The declaration of Helsinki. Appl. Psychophysiol. Biofeedback. **26**(1), 23–37 (2001). 10.1023/A:1009563504319 11387859 10.1023/a:1009563504319

[56] Dalia, D., Huseyin, N., Oriana, C., Sylwia, B.: Raising the bar for using surrogate endpoints in drug regulation and health technology assessment. BMJ. **374**, n2191 (2021). 10.1136/bmj.n2191 34526320 10.1136/bmj.n2191

[57] Single, A., Cabrera, A., Fifer, S., Tsai, J., Paik, J.-Y., Hope, P.: Patient advocacy group involvement in health technology assessments: An observational study. Res. Involv. Engagem. **7**(1), 83 (2021). 10.1186/s40900-021-00327-5 34823610 10.1186/s40900-021-00327-5 PMC8613914

[58] NICE: NICE health technology evaluations: the manual. (2022). https://www.nice.org.uk/process/pmg36/chapter/committee-recommendations#assessing-the-evidence

[59] Michaels, J.A.: Is NICE losing its standing as a trusted source of guidance? BMJ 383, p2571 (2023). 10.1136/bmj.p2571 10.1136/bmj.p2571 37940185

[60] NICE: Public Involvement Programme - Overview of technology appraisals: A factsheet for patient and carer organisations. In. National Institute for Health and Care Excellence: (2014)

[61] NICE: Public Involvement Programme - Scoping technology appraisals: a factsheet for patient and carer organisations. (2015). https://www.nice.org.uk/Media/Default/About/NICE-Communities/Public-involvement/Developing-NICE-guidance/Scoping-Technology-Appraisals-Factsheet-Patient-Carer.pdf

[62] NICE: Public Involvement Programme - Hints and tips for nominating patient experts: a factsheet for patient and carer organisations. (2015). https://www.nice.org.uk/Media/Default/About/NICE-Communities/Public-involvement/Developing-NICE-guidance/Nominating-Patient-Experts.pdf

[63] NICE: Patient Expert Submission Template. (2023). https://www.nice.org.uk/Media/Default/About/NICE-Communities/Public-involvement/Developing-NICE-guidance/Patient-expert-statement-template.docx

